# Is simultaneous bilateral unicompartmental knee arthroplasty and total knee arthroplasty better than simultaneous bilateral total knee arthroplasty?

**DOI:** 10.1186/s43019-023-00183-5

**Published:** 2023-04-27

**Authors:** Naosuke Nagata, Takafumi Hiranaka, Koji Okamoto, Takaaki Fujishiro, Toshikazu Tanaka, Anjiki Kensuke, Daiya Kitazawa, Ken Kotoura

**Affiliations:** Department of Orthopedic Surgery and Joint Surgery Centre, Takatsuki General Hospital, 1-3-13, Kosobe-Cho, Takatsuki, Osaka 569-1192 Japan

**Keywords:** Unilateral knee arthroplasty, Total knee arthroplasty, Simultaneous bilateral arthroplasty, Surgical invasion, Complication, One side

## Abstract

**Introduction:**

This retrospective study aims to clarify if there are benefits of performing unicompartmental knee arthroplasty (UKA) on just one indicated side in patients who undergo simultaneous bilateral knee arthroplasty.

**Materials and methods:**

We compared 33 cases of simultaneous bilateral UKA/total knee arthroplasty (TKA) (S-UT) with 99 cases of simultaneous bilateral TKA (S-TT). Comparison included blood tests [C-reactive protein (CRP), albumin, and D-dimer], the incidence of deep vein thrombosis (DVT), range of motion (ROM), and clinical scores before and 1 year after surgery.

**Results:**

Clinical scores were not significantly different between the groups. The postoperative flexion angle was significantly better in UKA sides. Blood tests showed that the S-UT had a significantly higher albumin value at 4 and 7 days after surgery. The CRP value at 4 and 7 days, and the D-dimer value at 7 and 14 days after surgery were significantly lower in the S-UT. The S-UT had significantly lower incidence of DVT.

**Conclusions:**

In cases of bilateral arthroplasty, if there is an indication on only one side, a better flexion angle can be obtained by UKA on that side, and with less surgical invasion. Moreover, the incidence of DVT is low, which is considered to be a benefit of performing UKA on just one side.

## Introduction

Simultaneous bilateral arthroplasty usually equals simultaneous bilateral total knee arthroplasty (S-TT) or simultaneous bilateral unicompartmental knee arthroplasty (S-UU), but sometimes unicompartmental knee arthroplasty (UKA) is only indicated on just one side. Many reports have compared unilateral UKA with unilateral total knee arthroplasty (TKA) or bilateral UKA with bilateral TKA, and there has been indication that UKA had more advantages than TKA [1–8]. Meanwhile, there are comparatively few reports of simultaneous bilateral UKA/TKA (S-UT) in the same patients [5, 6, 8–10] and it has been unclear whether S-UT has any advantages over S-TT. S-UT and S-TT have not been properly compared.

This study therefore aims to prove that the same surgical procedure is not necessarily required on both sides and that there may be advantages to performing UKA on only one side in bilateral simultaneous arthroplasty. With this background, and with a comparative lack of studies, we hypothesized that S-UT could be superior to S-TT in terms of blood examinations, functions, and complications.

## Materials and methods

### Patients

This retrospective study was approved by the institutional review board of our hospital. We performed knee arthroplasty for 1197 patients in our hospital between January 2013 and April 2018. Of these, simultaneous bilateral arthroplasty was performed for 548 patients, S-TT for 234 patients, S-UU for 276 patients, and S-UT for 38 patients. The study included 33 consecutive patients that underwent simultaneous S-UT. Five patients were excluded: three because they underwent lateral UKA and two because sufficient records were unavailable. We also selected 99 patients that underwent simultaneous S-TT. Patients were excluded if they underwent patella–femoral arthroplasty or if they had insufficient records. Patients who were hospitalized for more than 2 weeks were excluded from this study because they likely had complications. On the UKA side, Oxford Partial Knee (Zimmer Biomet, Warsaw, IN) mobile-bearing UKA was always used. On the TKA side, the cruciate-retaining (CR) knee TKA (Persona CR, NexGen CR-Flex or Vanguard CR Zimmer Biomet, Warsaw, IN) were always used. Indication for arthroplasty was osteoarthritis with full thickness cartilage loss of at least one compartment with continuous pain and loss of function, despite conservative treatment for at least 3 months [18]. The suitability of the UKA was evaluated in respective knees. If the knee fit the indication for UKA, UKA was performed, otherwise TKA was performed.

### Candidates for UKA

Candidates for UKA were selected based on radiological decision aids (the manual provided by the manufacturer) [19], with diagnosis of anterior medial type OA by preoperative radiographs [20]. Anteromedial OA was indicated by bone-on-bone arthritis in the medial compartment on varus stress radiographs. Intact lateral joint space and correctable varus deformity on valgus stress radiographs were required to demonstrate that there was full-thickness cartilage in the lateral compartment, acceptable patellofemoral disease (without lateral bone loss, grooving, or subluxation), and no extension of tibial erosion to the back of the tibial plateau on lateral radiographs. Knees with flexion contracture > 15° or flexion < 90° were also considered to be contraindications. If preoperative judgment was difficult, TKA was prepared as backup, and the type of arthroplasty was selected intraoperatively. All procedures were performed by the senior author or under his direct instruction.

### Treatment after the operation

Immediately after the operation, elastic stockings and foot pumps were applied on both legs. The following day, full weight bearing was encouraged, and rehabilitation was carried out by a physiotherapist. Edoxaban (15 mg/day) was orally administered for 14 days after surgery. If a patient already took antiplatelet drugs, they were restarted the day after the surgery instead of edoxaban. In general, the length of hospital stay was 2 weeks.

### Blood examinations

To determine the level of surgical invasion, we performed blood examinations including serum albumin (Alb) score and C-reactive protein (CRP) scores before, and then 1, 4, and 7 days after the operation, and the D-dimer score was determined 7 and 14 days after the operation.

### Clinical outcomes

We determined the active range of motion and clinical scores [Oxford Knee Score (OKS), Knee Society Score (KSS), and KSS function score] before and 1 year after surgery, and complications with or without deep vein thrombosis (DVT). Examination with lower limb vascular echo determined whether patients with high D-dimer score had other indications, such as Hohmann’s sign of lower leg DVT. If thrombus was found in the veins of the lower extremities regardless of mobility, the patient was considered to have DVT. DVT evaluation by lower limb echo was not performed before surgery.

### Statistical analysis

A description of measures was performed using means ± standard deviation (SD). Independent *t*-tests and Pearson’s Chi-square tests were used to compare the S-UT and S-TT groups. A paired *t*-test was used to compare range of motion (ROM) of the UKA and TKA knees before and after surgery. To adjust the preoperative flexion angle between the groups, linear regression analysis and analysis of covariance (ANCOVA) were used. Pearson’s correction coefficient was used to evaluate the relationships between pre- and postoperative flexion angles. All statistical analyses were performed using EZR (Easy R) (https://www.jichi.ac.jp/saitama-sct/SaitamaHP.files/statmed.html) [21].

## Results

### Patient characteristics

No significant differences were found in age, gender, or body mass index (BMI) of the patients (Table 1).

**Table 1:** Characteristics of the patients in the UT group and TT group

	UT group (33 cases)	TT group (89 cases)	*P*-value
Gender	25 Females (76%), 8 males (24%)	78 females (88%), 11 males (12%)	0.11
Age (years)	74.4 ± 8.0 (47–91)	74.7 ± 7.9 (58–87)	0.85
BMI (kg/m^2^)	25.9 ± 4.6 (18–42)	25.2 ± 3.8 (19–32)	0.45

### Blood examinations

Details of blood tests are presented in Table 2. The mean value of preoperative serum Alb was significantly higher in the S-UT group compared with in the S-TT group. On days 1, 4, and 7 after surgery, the values were significantly higher in the S-UT group. Regarding the CRP, the values were significantly lower in the S-UT group on days 4 and 7, with the peak on day 4 after surgery. The D-dimer value was significantly lower in the S-UT group on both day 7 and day 14 after surgery.

**Table 2:** Comparison of blood tests (albumin, CRP, D-dimer) between UT and TT Group

Blood tests		UT group(33cases)	TT group(89cases)	*P*-value	Cohen’s d*
Albumin (g/dl)	Preoperation	4.3 ± 0.3	4.1 ± 0.4	0.01	0.48
	Day 1	3.4 ± 0.3	3.3 ± 0.4	0.03	0.44
	Day 4	3.0 ± 0.4	2.8 ± 0.4	0.004	0.59
	Day 7	3.3 ± 0.4	3.1 ± 0.4	0.003	0.61
CRP (mg/dl)	Day 1	4.5 ± 2.3	4.6 ± 2.7	0.8	–
	Day 4	10.7 ± 4.8	12.9 ± 5.1	0.03	0.43
	Day 7	3.8 ± 3.0	5.1 ± 3.3	0.04	0.41
D-dimer	Day 7	13.2 ± 5.3	17.4 ± 7.1	0.002	0.62
	Day 14	12.2 ± 6.0	17.6 ± 9.3	0.002	0.62

*Cohen’s d: effect size (0.20 = small, 050 = medium, 0.80 = large)

### Complications

The incidence of DVT was significantly lower in the S-UT group. There were 41 of 99 cases (41.4%) in the S-TT group compared with 7 of 33 cases (21.2%) in the S-UT group (*P* = 0.04). Other complications, such as postoperative infection, perioperative fracture, and readmission within 30 days, were not observed in either group.

### Range of motion

**Fig. 1:**
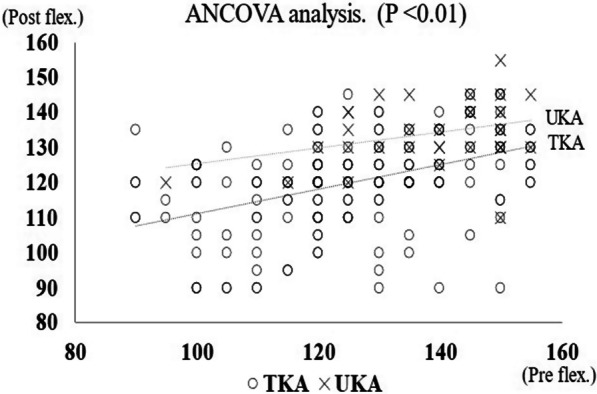
Analysis of covariance which the postoperative flexion angle was corrected by the preoperative flexion angle

Details of the range of motion are presented in Table 3. Preoperatively, UKA knees were significantly better than TKA knees in both extension and flexion. Postoperatively, the extension angle improved in both UKA and TKA knees, with no significant difference between them. By contrast, the flexion angle was significantly deeper in UKA knees than in TKA knees. Analysis of covariance (ANCOVA), in which the postoperative flexion angle was corrected by the preoperative flexion angle, also showed significantly better values for the UKA knees (Fig. 1).

**Table 3:** Comparison of range of motion between UKA and TKA Group

ROM°		UKA	TKA	*P* value	Cohen's d*
pre-op	Extension	− 3.8 ± 5.3	− 8.2 ± 8.1	0.002	0.57
	Flexion	138.5 ± 13.6	124.3 ± 19.9	< 0.001	0.74
post-op	Extension	0.8 ± 2.5	− 0.5 ± 2.1	0.52	–
	Flexion	133.9 ± 9.2	118.4 ± 15.0	< 0.001	1.08

*Cohen’s d: Effect size (0.20 = small, 050 = medium, 0.80 = large)

### Clinical outcomes

In the S-UT group, OKS and KSS function score all had preoperative to postoperative improvement. Similarly, improvement was observed in each score in the S-TT group. No significant differences were found, however, between the S-UT group and the S-TT group before and after surgery. KSS was used for clinical score comparison between UKA knees and TKA knees. Preoperative UKA knees were 60.8 ± 16.4, while TKA knees were 58.1 ± 16.5, showing no significant difference. Postoperative UKA knees were 88.0 ± 10.9, while the TKA knees were 90.2 ± 9.5, showing improvement in the score in both groups, but without significant difference. Clinical outcomes are presented in Table 4.

**Table 4:** Comparison of pre- and postoperative clinical outcomes between UT and TT groups

	UT group	TT group	*P*-value
OKS			
Preoperation	24.2 ± 8.5	24.1 ± 7.9	0.93
Postoperation	34.8 ± 7.3	36.1 ± 7.4	0.39
KSS function score			
Preoperation	53.8 ± 23.7	52.2 ± 23.6	0.73
Postoperation	72.6 ± 15.7	72.8 ± 16.8	0.94

## Discussion

The most important finding of this study was that there were benefits of simultaneous bilateral knee arthroplasty by performing UKA if there was an indication on just one side.

The blood sampling results and complication rates show that surgery was less invasive with UKA, even on one side. There have been many reports, some of which are detailed below, on one-sided and two-sided simultaneous UKA or TKA, but no report has compared S-UT and S-TT until now.

### Complications

There are various reports, for example, on the incidence of DVT. Chan et al. reported that the incidence of DVT in S-UU was clearly higher in one-stage bilateral surgery than in staged bilateral surgery [22]. However, Duchman et al. compared TKA with UKA and found that UKA had a significantly lower incidence of DVT [23]. Kim et al. reported that there was no significant difference in the incidence of DVT between S-TT and unilateral TKA [24]. Many reports have stated that the incidence of distal DVT was relatively high, but there were few fatal complications if thromboprophylaxis were taken. Sueta et al. reported no proximal DVT, irrespective of whether edoxaban was used after TKA, but the incidence of distal DVT was significantly lower in the oral group compared with in the non-oral group (Table 5) [25]. In the current study, no pulmonary embolism (PE) was found in either group, and the incidence of asymptomatic distal DVT was significantly lower in the S-UT group compared with in the S-TT group. Prevention of DVT with edoxaban has been successful, so it cannot be said that simultaneous bilateral surgery should be avoided. In addition, if UKA is indicated, there is a benefit in that the incidence of DVT can be reduced by selecting UKA on only one side in bilateral surgery.

**Table 5:** Comparative studies of DVT incidence

	Incidence of DVT		*P*-value
Chan et al.	One-stage bilateral UKA	Staged bilateral UKA	–
	10/159 (6.3%)	3/80 (3.8%)	
Duchman et al.	UKA	TKA	0.0186
	0.50%	1.50%	
Kim et al.	Bilateral TKA	Unilateral TKA	–
	7/2358 (0.3%)	6/719 (0.8%)	
Sueta et al.	TKA with edoxaban	TKA without edoxaban	0.038
	3/19 (15.8%)	10/19 (52.6%)	
This study	UT group	TT group	0.04
	7/33 (21.2%)	41/99 (41.4%)	

### Blood examinations

We examined Alb and CRP levels as indicators of surgical invasiveness. These are considered to be acute phase reactants, and inflammation in the body causes a decrease in Alb and an increase in CRP [26, 27]. Significant differences in Alb levels were observed before and on days 1, 4, and 7 after surgery. As far as we are aware, no other papers have described the degree of surgical invasion regarding preoperative and postoperative serum Alb levels. The reason for preoperative Alb level being significantly lower in the S-TT group is that although there are no scores that show a significant difference in preoperative activities of daily living, it is thought that the activity is lower in patients in the S-TT group. The nutritional status of patients is said to be related to postoperative results and infection rate in orthopedic surgery [28, 29], but in this study, there were no cases with postoperative complications. After surgery, Alb levels were significantly higher in the S-UT group, suggesting that UKA was less invasive. CRP levels were significantly lower in the S-UT group on days 4 and 7 after surgery. Tanaka et al. stated that the CRP levels at 1 week after surgery were significantly lower in UKA, so when comparing unilateral UKA and unilateral TKA, UKA was less surgically invasive [30].

High D-dimer levels are an indicator of increased fibrinolysis of intravascular thrombus. The D-dimer level was significantly lower in the S-UT group on both day 7 and day 14 after surgery. Tanaka et al. compared UKA and TKA, with significantly lower D-dimer values 1 and 2 weeks after surgery in UKA, suggesting that UKA is less invasive [30]. From these blood tests, it can be said that there are benefits in selecting UKA if there is an indication for UKA on only one side.

### Range of motion

In comparison of UKA and TKA within the same S-UT patient, Dalury et al. and Laurencin et al. reported that UKA knees had a significantly better flexion angle after surgery than TKA knees [5, 6]. After adjustment for preconditions, Hiranaka et al. also reported that UKA knees had a significantly better flexion angle than TKA knees in a comparison of UKA and TKA within the same S-UT patients [10]. In our study, UKA had significantly better ROM than TKA. However, both UKA and TKA knees showed a mild decrease in postoperative flexion angle, with no significant difference for UKA knees (*P* = 0.065) and a significant difference for TKA knees (*P* < 0.001). Although knee arthroplasty reportedly improves postoperative ROM, these reports did not take preoperative ROM into account. In other words, it does not make sense to compare pre- and postoperative ROM itself, but rather to consider ROM or the change of ROM by correcting for the preoperative values [10]. In the present study, we corrected for preoperative ROM by performing an analysis of covariance and found that for both UKA and TKA, the worse the preoperative ROM, the better the postoperative ROM. Although mild decrease in postoperative flexion angle was observed, it might be due to a better preoperative flexion angle than in other reports.

From the above results, UKA is suggested to have a better postoperative flexion angle than TKA, which leads to improvement of activities of daily living. If there is an indication for UKA on only one side in bilateral simultaneous arthroplasty, there are benefits of using UKA.

### Clinical outcomes

In a comparison of UKA and TKA in the same patient, previous studies reported no significant difference in clinical scores [6, 8]. In a comparison of S-UU with staged bilateral UKA, Berend et al. reported that simultaneous bilateral cases had significantly better scores in terms of KSS function [16]. In the current study, there was no significant difference between the two groups in OKS and KSS function score both before and after surgery.

Our report focuses upon short-term results 1 year after surgery, so the long-term results remain unknown. As in the other reports, however, even in the case of S-UT, the same good results as bilateral S-TT were obtained. The same surgical procedure is not necessarily required on both sides: if there is an indication for UKA on only one side, using UKA on that side only has the benefit of minimal invasiveness.

### Limitations

This study has some limitations. First, it is a retrospective study, and the number of cases is small, so adjustment for patient backgrounds is required for statistical analysis. Second, there is very little literature on UKA/TKA within the same patient, so this report is in itself useful, but it is also difficult to make a comparative study. In Japan, postoperative hospitalization is often long term, about 2 weeks. Conversely, blood tests are more rare in the USA and Europe because of the comparatively short-term hospitalization. Blood test findings in this study are therefore difficult to compare with those of other studies. Third, the observation period was up to 1 year after surgery, so none of the cases were revision cases, but the possibility of revision will likely increase by extending the observation period.

### Conclusions

In a comparison of the S-UT and S-TT groups, clinical scores were not significantly different from the one-sided comparison. UKA was shown to be less invasive if there is an indication on only one side according to blood tests and incidence of DVT. Range of motion was significantly better in UKA knees than in TKA knees. Using UKA on only one side has benefits if there is indication for UKA in the case of simultaneous bilateral knee arthroplasty and the same surgical procedure is not necessarily required on both sides.

## Data Availability

Not applicable.
